# Carbon Nitride Decorated Ball-Flower like Co_3_O_4_ Hybrid Composite: Hydrothermal Synthesis and Ethanol Gas Sensing Application

**DOI:** 10.3390/nano8030132

**Published:** 2018-02-27

**Authors:** Yuxiao Gong, Yan Wang, Guang Sun, Tiekun Jia, Lei Jia, Fengmei Zhang, Long Lin, Baoqing Zhang, Jianliang Cao, Zhanying Zhang

**Affiliations:** 1 School of Chemistry and Chemical Engineering, Henan Polytechnic University, Jiaozuo 454000, China; gyx201311@163.com (Y.G.); jlxj@hpu.edu.cn (L.J.); mei1994w@163.com (F.Z.); linlong@hpu.edu.cn (L.L.); bqzhang@hpu.edu.cn (B.Z.); zhangzy@hpu.edu.cn (Z.Z.); 2 The Collaboration Innovation Center of Coal Safety Production of Henan Province, Jiaozuo 454000, China; 3 State Key Laboratory Cultivation Bases Gas Geology and Gas Control (Henan Polytechnic University), Jiaozuo 454000, China; 4 Department of Materials Science and Engineering, Luoyang Institute of Science and Technology, Luoyang 471023, China; tiekunjia@126.com

**Keywords:** carbon nitride, ball-flower like Co_3_O_4_, nanocomposites, ethanol, gas sensor

## Abstract

Recently, semiconducting metal oxide (SMO) gas sensors have attracted the attention of researchers for high conductivity, labile features by environment, low cost, easy preparation, etc. However, traditional SMOs have some defects such as higher operating temperature and lower response value, which greatly limit their application in the field of gas sensor. In this work, the carbon nitride decorated ball-flower like Co_3_O_4_ composite was successfully synthesized via a facile hydrothermal method, the composition and morphology of the as-synthesized samples were studied by the techniques of X-ray powder diffraction (XRD), Field-emission scanning electron microscopy (FESEM), Transmission electron microscopy (TEM), Fourier transform infrared spectrometer (FT-IR) and N_2_-sorption. As a consequence, the pure Co_3_O_4_ and the carbon nitride decorated Co_3_O_4_ both possess ball-flower like structure, and the as-synthesized carbon nitride decorated Co_3_O_4_ composite exhibits significant sensing properties to ethanol which is 1.6 times higher than that of pure Co_3_O_4_, furthermore, the composite possesses high selectivity and stability towards ethanol detection.

## 1. Introduction

In the last decade, with the prosperity of the decoration market, “paint” has been widely concerning due to the harmful, volatile organic compound (VOC) that it produces. In the present study, noble metals (Au, Ag) are sensitive to gas, but they have high price, poor stability [1] (for the increasing particles affected by light, humidity, the accumulating carbonate on the surface of Au particles) and a short lifetime, making their application in gas sensors difficult to apply in industrial production and daily life. Thus, SMOs are widely researched and applied in the gas sensor to detect hazardous and toxic gases owing to their high conductivity, labile features by environment, low cost, and easy preparation, for instance, the ZnO [2,3], SnO_2_ [4,5], CuO [6], Co_3_O_4_ [7,8], α-Fe_2_O_3_ [9], NiO [10], In_2_O_3_ [11], and WO_3_ [12,13]. SMOs have defects, such as high operating temperature, low sensitivity, and poor selectivity. The key to improving the response and operating temperature of gas sensors is selecting suitable material and modifying traditional SMOs.

In general, Co_3_O_4_ is an important p-type semiconductor with rich oxygen content, and is widely used in fields such as lithium-ion batteries, catalysts, and supercapacitors [14]. The researchers also apply Co_3_O_4_ in the field of gas sensors due to its good chemical stability and high specific surface area. For example, Patil et al. reported that Co_3_O_4_ nanorods had high sensitivity and fast response to CO [15]. Li et al. showed that Co_3_O_4_ nanotubes possess the property of high response to H_2_ and C_2_H_5_OH [7]. Hoa Nguyen et al. synthesized the porous Co_3_O_4_ nanorods, which showed good stability, fast response and recovery time when it was used as the gas sensor [8]. Researchers synthesized Co_3_O_4_ with other negative ions or metal oxides. Davide et al. improved p-type sensing materials by fluorine-doped Co_3_O_4_ nanosystems, thereby enhancing reversibility and lowering the working temperature [16]. Zhang et al. synthesized acicular nanowire of TiO_2_ decorated Co_3_O_4_ with high response and low operating temperature [17].

Recently, two-dimensional (2D) nanomaterials have constituted an important domain of nanostructures and were widely used in the field of gas sensors, due to their high specific surface area, unique electronic structure and excellent chemical inertness [18,19,20,21]. Wang et al. synthesized holey reduced graphene oxide nanosheets with high sensor response and reversible sensing for NH_3_ detection [22]. Cao et al. improved sensing properties through g-C_3_N_4_ nanosheets decorated SnO_2_ composites toward ethanol gas [23]. Chen et al. reported that Co_3_O_4_-rGO nanoparticles showed a good response and recovery to methanol [24]. Cao et al. synthesized the SnO_2_/g-C_3_N_4_ composites with high performance to ethanol via a facile calcination method [25]. Actually, reports on Co_3_O_4_ as gas sensor are limited, and there is no report on Co_3_O_4_ decorated pCNH composite for gas sensor application. The polymeric to graphitic carbon nitride (pCNH) [26,27] is one of the classes of carbon nitrides formed by themolysis and other reactions, which is a polymeric amorphous soild containing a high concentration of H component, and based on ribbon-like polyheptazine structural units like Liebig’s melon.

In this paper, we report the synthesis of pCNH decorated Co_3_O_4_ composite (Co_3_O_4_/pCNH) via a simple hydrothermal method. The presence of ball-flower like Co_3_O_4_ and pCNH is characterized by the techniques of XRD, FESEM, TEM, FT-IR, and N_2_-sorption, and the gas sensing performance of the prepared composite is investigated by exposing to various concentration of ethanol vapor at different temperature. As a result, the Co_3_O_4_ and Co_3_O_4_/pCNH composite possesses ball-flower like structure, and the Co_3_O_4_/pCNH composite exhibits excellent performance, such as higher sensor response, excellent selectivity and stability toward ethanol vapor.

## 2. Results and Discussion

### 2.1. Sample Characterization

**Figure 1:**
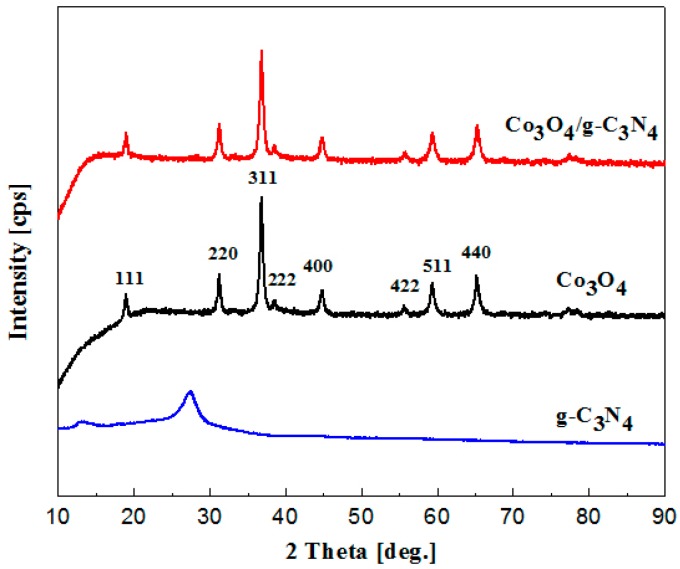
XRD patterns of pCNH, Co_3_O_4_ and Co_3_O_4_/pCNH composite.

**Figure 2:**
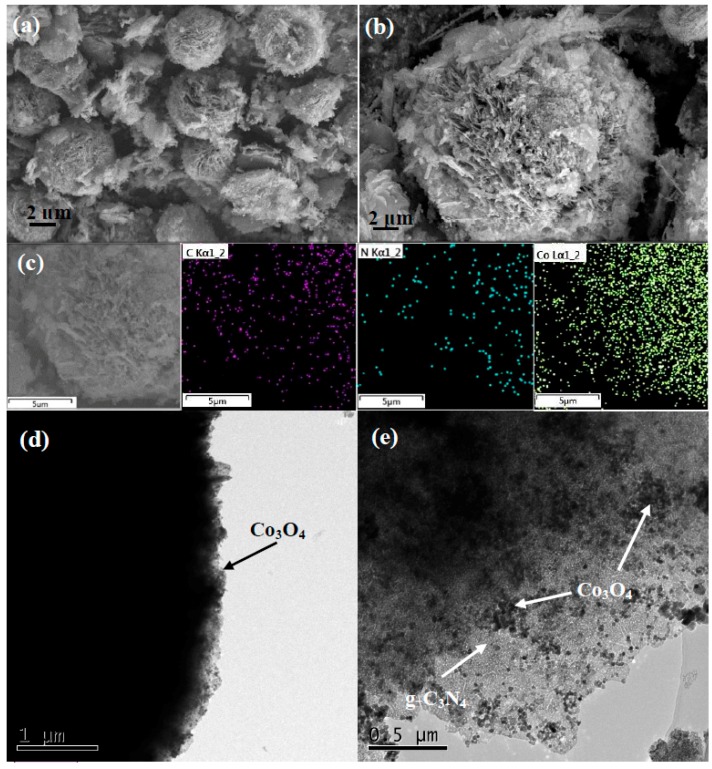
FESEM images of pure Co_3_O_4_ (**a**); Co_3_O_4_/pCNH composite (**b**); EDS element mappings of Co_3_O_4_/pCNH composite (**c**); and TEM images of pure Co_3_O_4_ (**d**) and Co_3_O_4_/pCNH composite (**e**).

Figure 1 shows the typical patterns of pCNH, Co_3_O_4_ and Co_3_O_4_/pCNH composite. From the typical XRD patterns, the phase purity and crystallinity are clearly characterized, and Figure 1 shows two diffraction peaks at 12.9° and 27.5° of the pCNH corresponding to the (100) and (002) crystal planes. The peak at 12.9° of pCNH is the inter-layer structural packing. The highest peak at 27.5° corresponds to the characteristic inter planar staking peak of aromatic systems. The main diffraction peaks are located at 2θ of 19.0°, 31.2°, 36.8°, 38.5°, 44.8°, 55.6°, 59.3° and 65.2° corresponding to the (111), (220), (311), (222), (400), (422), (511) and (440) crystal planes, respectively [28,29,30]. All the peaks of the pure Co_3_O_4_ can be assigned to Co_3_O_4_ (JCPDS No. 42-1467). The peak intensity is strong indicting high crystalline structure of the products. No peak from other phases is detected, indicating high purity of the products. For the Co_3_O_4_/pCNH composite, it is hard to find the characteristic peak of pCNH for two reasons [31,32,33,34]. On the one hand, the quantity of pCNH in Co_3_O_4_/pCNH composite is small. Moreover, the X-ray diffraction has a limit of detection which contributes to the hard observation of the pCNH peak in XRD patterns. On the other hand, the cobalt ions can be absorbed onto the surface of the pCNH sheets through electrostatic attraction and the in situ formed Co_3_O_4_ might attach to the surfaces of pCNH nanosheets and prevent their aggregation and restacking, which can weaken the characteristic peak of pCNH [34,35,36]. One can see from the Energy Dispersive Spectrometer (EDS) element mappings of Co_3_O_4_/pCNH composite (Figure 2c) that the C, N and Co elements are simultaneously detected and these elements are highly dispersed in the composite, providing the coexistence of Co_3_O_4_ and pCNH in the as-prepared sample. Further evidence for this conjectural can be supported by TEM (Figure 2e).

**Figure 3:**
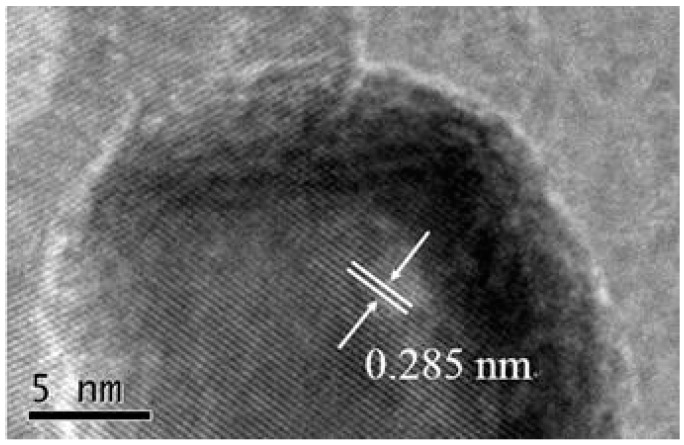
HRTEM image of the Co_3_O_4_/pCNH composite.

The morphology and microstructure of the obtained samples are verified by using the techniques of FESEM, EDS and TEM. As shown in Figure 2a, the pure Co_3_O_4_ possesses the hierarchical and ball-flower like structure with diameters range of 5–7 μm, and the ball-flower like structure is composed of a number of Co_3_O_4_ nanosheets. From Figure 2b, we can see that the Co_3_O_4_/pCNH composite keeps the ball-flower like structure which is similar to the pure Co_3_O_4_, and Co_3_O_4_ and pCNH is closely linked together. Figure 2c shows the EDS element mappings of Co_3_O_4_/pCNH composite, from Figure 2c one can see that the C, N and Co elements are simultaneously detected and these elements are highly dispersed in the composite, further providing the coexistence of Co_3_O_4_ and pCNH in the as-prepared sample. We can see from the TEM image of pure Co_3_O_4_ (Figure 2d) that it possesses the ball-flower like structure and the Co_3_O_4_ nanosheet is composed of a number of nanoparticles. Figure 2e displays the typical TEM image of the Co_3_O_4_/pCNH composite. The morphology and microstructure of pCNH has been verified with thin layers in our previous paper [25]. It can be seen from Figure 2e that polymeric to graphitic carbon nitride (pCNH) support the Co_3_O_4_ particles, and the Co_3_O_4_ nanoparticles are highly dispersed on the surface of pCNH nanosheet, and the high surface area of pCNH nanosheet can suppress the sintering of Co_3_O_4_ nanoparticles. Due to the high dispersion of Co_3_O_4_ nanoparticles, the target gas molecules can immigrate and the produced molecules can emigrate easily from the surface; the as-prepared ball-flower like structure composite is believed to achieve high gas sensing performance. The high-resolution transmission electron microscopy (HRTEM) of the as-prepared Co_3_O_4_/pCNH is shown in Figure 3. From this figure, the plane spacing of 0.285 nm corresponds to the lattice planes of (220) in Co_3_O_4_. This result could confirm that the nanoparicles visible are Co_3_O_4_ phase on the surface of pCNH nanosheet.

**Figure 4:**
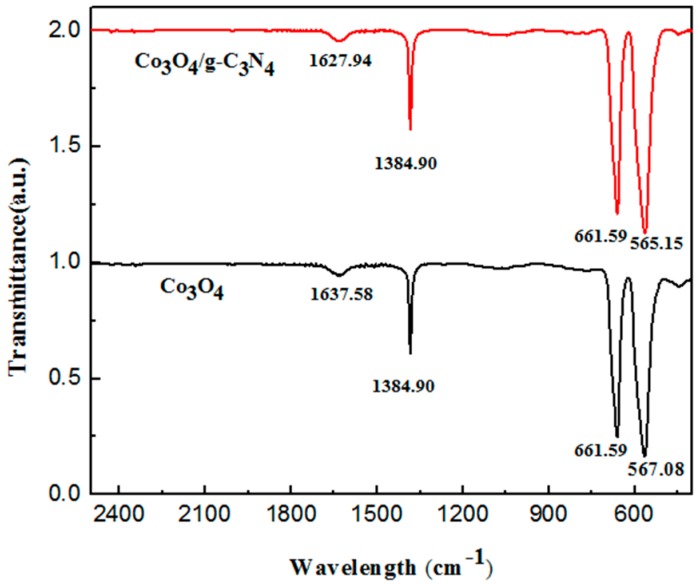
FT-IR spectra of Co_3_O_4_ and Co_3_O_4_/pCNH composite.

Figure 4 shows the FT-IR spectrum of pure Co_3_O_4_ and the Co_3_O_4_/pCNH composite. The FT-IR spectrum of Co_3_O_4_/pCNH composite has no new absorption peak showing that the structure of Co_3_O_4_ and pCNH in the compound has not been destroyed. The peak at 1384.90 cm^−1^ of Co_3_O_4_ is due to the C–H bending vibration, the sharp peak at 567.08 cm^−1^ and 661.59 cm^−1^ of pure Co_3_O_4_ are attributed to Co–O stretching vibration modes. However, the Co–O stretching vibration modes of the Co_3_O_4_/pCNH composite are at 565.15 cm^−1^ and 661.59 cm^−1^. These bands experience an offset at around 560–570 cm^−1^ [37]. The bands at 1637.58 cm^−1^ of pure Co_3_O_4_ are attributed to vibration of the C=C. Nevertheless, the vibration of the C=C of Co_3_O_4_/pCNH appears at 1627.94 cm^−1^. There produces a large deviation indicating an electronic transfer between the C=C and Co–O. The author thinks that there are bonding effects between the Co_3_O_4_ and pCNH for the in situ formed.

**Figure 5:**
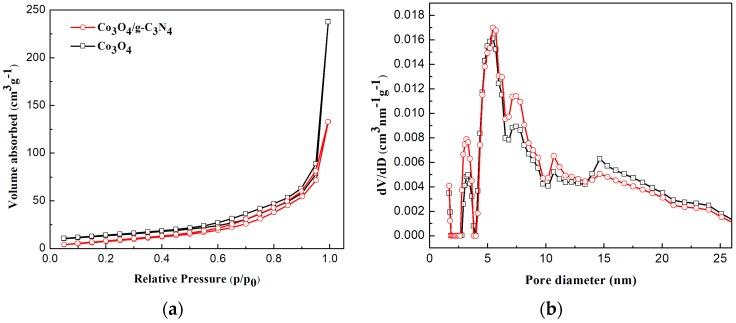
N_2_ adsorption-desorption isotherms (**a**) and the corresponding pore size distribution curves (**b**) of Co_3_O_4_ and Co_3_O_4_/pCNH composite.

Figure 5 displays the N_2_ adsorption-desorption isotherms and the corresponding pore size distribution curves of the as-prepared Co_3_O_4_ and Co_3_O_4_/pCNH composite. As shown in Figure 5a, the isotherms of the Co_3_O_4_ and Co_3_O_4_/pCNH composite is categorized to type IVaccording to the IUPAC, with the hysteresis loop of H_3_-type [2]. This not only shows that it is categorized typical mesoporous material but it also illustrates the existence of an aggregation of the laminated structure with a narrow slit formed by the pCNH and Co_3_O_4_ composites from hysteresis loop of H_3_-type. Figure 5b shows the corresponding pore size distribution curves of the Co_3_O_4_ and Co_3_O_4_/pCNH composite. It can be clearly seen that the pore diameter of Co_3_O_4_ and Co_3_O_4_/pCNH composite all main distribute upon 5.44 nm. The results reveal that these two samples are categorized to typical mesoporous materials and matching well with the interlayer pores observed by SEM and TEM image. The BET surface area of Co_3_O_4_ and Co_3_O_4_/pCNH composite correspondingly turned out to be 48.6 m^2^·g^−1^ and 51.3 m^2^·g^−1^, respectively. The specific surface area of Co_3_O_4_/pCNH composite has a certain improvement over pure Co_3_O_4_. The improvement can affect the activity of materials, and in turn, enhancing the gas-sensing performance.

### 2.2. Gas-Sensing Performance

**Figure 6:**
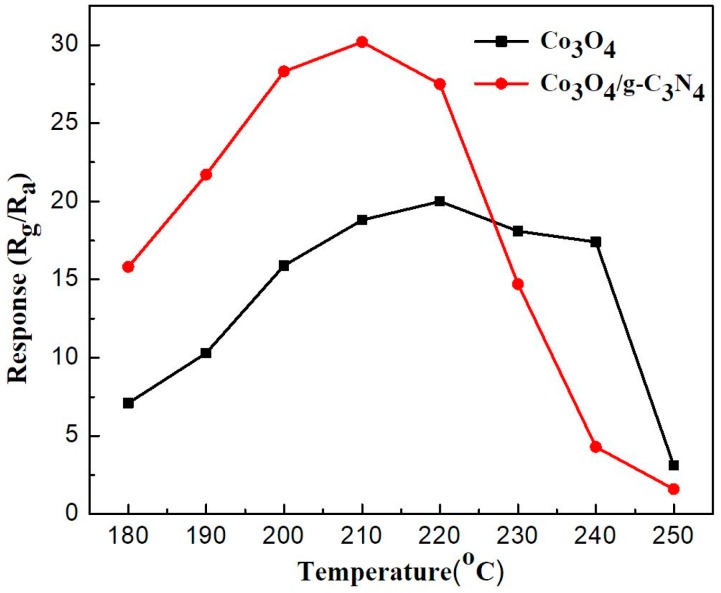
Response values of the sensors based on Co_3_O_4_ and Co_3_O_4_/pCNH composite toward 500 ppm ethanol vs. operating temperature.

In order to investigate the gas sensing performance of the as-synthesized samples-based sensors to ethanol vapor, a series of tests are performed. In this study, during the gas sensing performance test, 500 ppm of ethanol is introduced into a sealed chamber (the relative humidity is 40% in the test chamber). The relative humidity can be observed on an intelligent gas sensing analysis system of CGS-4TPS (Beijing Elite Tech. Co., Ltd., Beijing, China). In order to keep humidity at around 40%, the work was continued without intermittence and completed in a day. There is a preparation of seeking optimal temperature from room temperature to high temperature before the formal work; the preparation work shows optimal temperature at around 210 °C. As a result, the formal work sets 180–250 °C as a test range and re-tests to seek optimal temperature. Gas sensing parameters are studied, such as operating temperature, gas response, response-recovery time, and stability for gas sensors. Figure 6 shows the ethanol sensing properties of Co_3_O_4_ and Co_3_O_4_/pCNH composite based sensors with an ethanol concentration of 500 ppm, and a working temperature was in the range of 180–250 °C. The optimal operating temperatures of Co_3_O_4_ and Co_3_O_4_/pCNH composite based sensors are at 220 °C and 210 °C, respectively. The response value of Co_3_O_4_/pCNH composite based sensor to ethanol vapor at 210 °C is 30.2. However, this value of pure Co_3_O_4_ based sensor is only 18.8. In the author’s view, the increased response value of Co_3_O_4_/pCNH composite based sensor toward ethanol has two causes. On the one hand, the pCNH is supporting the Co_3_O_4_ particles, and increasing the surface area of Co_3_O_4_/pCNH composite, in turn, leading to more Co^3+^ as the extra adsorption centers for ethanol. On the other hand, the strong coupling observed by FT-IR spectrum between the Cobalt and oxygen ions in the Co_3_O_4_/pCNH composite makes the Co–O more ionic in nature, which enhances the gas-sensing properties [34].

**Figure 7:**
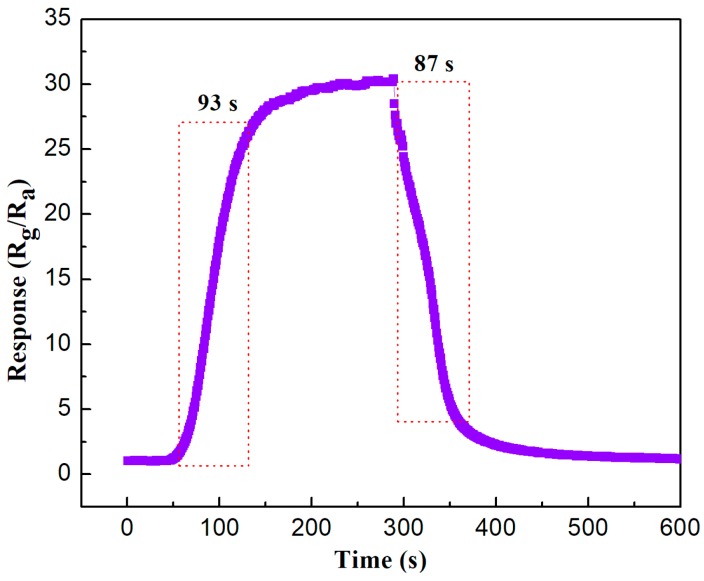
The response-recovery time of Co_3_O_4_/pCNH composite at 210 °C toward 500 ppm ethanol.

Figure 7 displays the response-recovery time of Co_3_O_4_/pCNH composite at 210 °C towards 500 ppm ethanol. Response time refers to the time taken as 90% by a gas sensor upon exposure to a target gas from the first reaction to the stable end value when the signal has reached a particular percentage level. Recovery time is the time required by a sensor so as to return to 90% of the original baseline signal when the target gas is removed and the sensor is subsequently cleaned with dry air. The response time of this Co_3_O_4_/pCNH composite is 93 s and the recovery time of this Co_3_O_4_/pCNH composite is 87 s towards 500 ppm ethanol at 210 °C.

**Figure 8:**
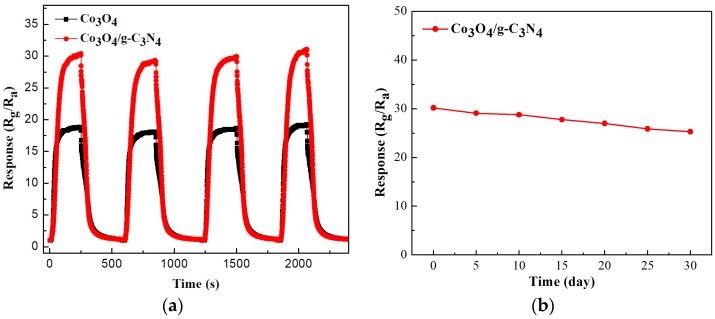
Therepeatability (**a**) and stability (**b**) of Co_3_O_4_ and Co_3_O_4_/pCNH composite based sensors toward 500 ppm at 210 °C.

Figure 8 shows the repeatability (a) and stability (b) of the pure Co_3_O_4_ and Co_3_O_4_/pCNH composite based sensor towards ethanol. It can be seen from Figure 8a that through repeating the test four times, the response recovery curves of the Co_3_O_4_/pCNH composite based sensor remain the original response value at around 30, which shows that the material has good repeatability. From Figure 8b, we can see that the response values maintain more than 80% of the original values after 30 days. So, we can draw the conclusion that the Co_3_O_4_/pCNH composite based sensor possesses an unexceptionable stability for ethanol vapor detection. The good repeatability and unexceptionable stability of the material make it possible to apply in the actual application.

**Figure 9:**
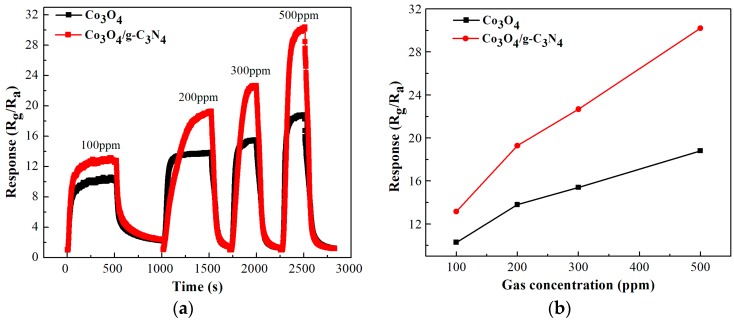
The real time response curves (**a**) and the response values (**b**) to different concentrations of ethanol of Co_3_O_4_ and Co_3_O_4_/pCNH composite based sensor at the operation temperature of 210 °C.

Figure 9 displays the response curves to different concentrations of ethanol of Co_3_O_4_ and Co_3_O_4_/pCNH composite based sensor. From Figure 9a, we can see that the response of the Co_3_O_4_ and Co_3_O_4_/pCNH goes up trend with the increase of ethanol vapor concentration. As shown in Figure 9b, there is the linear relationship between the response values and the concentration of ethanol for the Co_3_O_4_ and Co_3_O_4_/pCNH based sensor. The enhanced response values of the Co_3_O_4_/pCNH are more obvious with the increasing of gas concentration.

**Figure 10:**
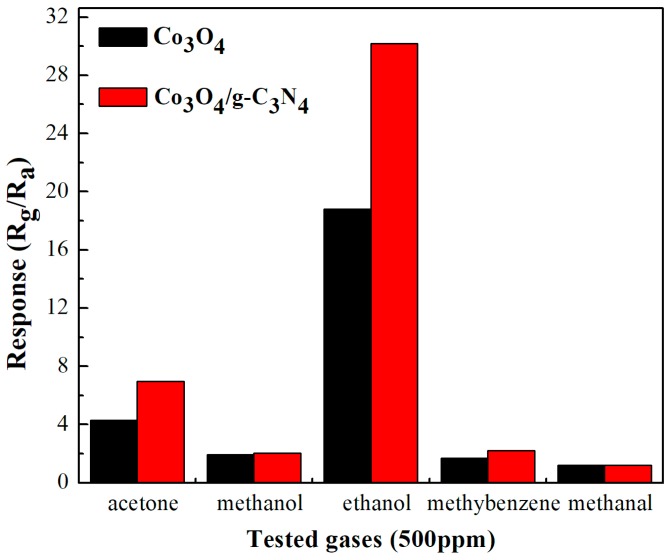
The selectivity of Co_3_O_4_ and Co_3_O_4_/pCNH composite based sensors toward 500 ppm different gases at the operation temperature of 210 °C.

The selectivity of obtained materials toward different gases is displayed in Figure 10. As shown in Figure 10, the response values of Co_3_O_4_/pCNH toward acetone, methanol, ethanol, methylbenzene, and methanol are 6.97, 2.04, 30.2, 2.2, 1.22, respectively. Apparently, the Co_3_O_4_/pCNH composite possesses the highest response toward ethanol vapor. Meanwhile, it also can be seen from Figure 10 that the properties of Co_3_O_4_/pCNH composite towards several gases are higher than the pure Co_3_O_4_, indicating that the Co_3_O_4_/pCNH composite has a potential application value for ethanol detection.

## 3. Materials and Methods

### 3.1. Sample Preparation

All chemical reagents are analytical and used without further purification. Polymeric to graphitic carbon nitride (pCNH) is synthesized by our previously reported method [38]. The synthesis of flower like pure Co_3_O_4_ follows our previous report method [39]. In a typical preparation process of carbon nitride decorated ball-flower like Co_3_O_4_ hybrid composite, 2.49 g Co(OAC)_2_·4H_2_O was first dissolved in 40 mL deionized water, 0.016 g pCNH was ultrasonic treated in 40 mL deionized water for 4 h. The two solutions were mixed, and then 6 mL of ammonia water was added slowly and stirred in the whole procedure. Then, the mixture was transferred into a 100 mL Teflon-lined stainless-steel autoclave and heated to 180 °C for 12 h under the autogenous pressure. After cooled to room temperature, the products were washed 3 times by deionized water and ethanol respectively to remove any possible impurities. Then, the obtained product was dried in a vacuum oven at 80 °C for 24 h. Finally, the powder was heated in a muffle furnace to 300 °C for 2 h at a heating rate of 2 °C·min^−1^. After naturally cooled to room temperature, the flower like Co_3_O_4_ and Co_3_O_4_/pCNH composite were obtained.

### 3.2. Characterizations

The samples were characterized by X-ray diffraction (XRD, Bruker-AXS D8, Bruker, Madison, WI, USA) with CuKα radiation at 40 kV and 25 mA. Fourier Transform Infrared Spectrometer (FT-IR) was recorded by Fourier Transform Infrared Spectrometer (TENSOR 27 Bruker plc, Madison, WI, USA). The morphology and structure of the samples were observed by the field-emission scanning electron microscopy (FESEM, Quanta™250 FEG) (FEI, Eindhoven, The Netherlands). Transmission electron microscopy (TEM) analysis was performed on a TecnaiG2 F20 microscope (FEI, Eindhoven, The Netherlands) operating at 200 kV. Nitrogen adsorption-desorption isotherms were obtained on a Quantachrome Autosorb-iQ sorption analyzer (Quantachrome, Boynton Beach, FL, USA).

### 3.3. Gas Sensor Fabrication and Analysis

Gas-sensing performance analysis of the as-synthesized sample was performed on an intelligent gas sensing analysis system of CGS-4TPS (Beijing Elite Tech. Co., Ltd., Beijing, China). The gas sensor fabrication method and gas sensing test produce were performed as our previously reported method [38]. Response of the gas sensor is defined as follows: Response = *R_g_*/*R_a_* (*R_a_* and *R_g_* are the resistances of the sensor measured in air and in test gas, respectively).

## 4. Conclusions

In summary, the ball-flower like Co_3_O_4_ and pCNH decorated ball flower-like Co_3_O_4_ composite (Co_3_O_4_/pCNH) are successfully synthesized via a facile hydrothermal route. The composition and morphology of the as-synthesized samples are studied by the techniques of XRD, FESEM, TEM, FT-IR and N_2_-sorption. The obtained Co_3_O_4_/pCNH composite shows good selectivity to ethanol, and the optimum temperature of Co_3_O_4_/pCNH composites is 210 °C. The response value of Co_3_O_4_/pCNH composite is 1.6 times higher than pure Co_3_O_4_ toward 500 ppm ethanol at 210 °C. The enhanced gas sensor properties are due to unique electronic structure and excellent substrate function of pCNH. The Co_3_O_4_/pCNH composite also shows a linear relationship between the response values and concentration, good repeatability and stability. The superior gas sensing properties of Co_3_O_4_/pCNH indicated that this research will be available for more applications in both laboratory and industry.

## References

[1] Konova P., Naydenov A., Venkov C., Mehandjiev D., Andreeva D., Tabakova T. (2004). Activity and deactivation of Au/TiO_2_, catalyst in CO oxidation. J. Mol. Catal. A Chem..

[2] Zeng B.R., Zhang L.C., Wu L.Q., Su Y.Y., Lv Y. (2017). Enclosed hollow tubular ZnO: Controllable synthesis and their high performance cataluminescence gas sensing of H_2_S. Sens. Actuators B.

[3] Kumar R., Aldossary O., Kumar G., Umar A. (2015). Zinc oxide nanostructures for NO_2_ gas–sensor applications: A review. Nano-Micro Lett..

[4] Wang B., Zhu L.F., Yang Y.H., Xu N.S., Yang G.W. (2008). Fabrication of a SnO_2_ nanowire gas sensor and sensor performance for hydrogen. J. Phys. Chem. C.

[5] Göpel W., Schierbaum K.D. (1995). SnO_2_ sensors: Current status and future prospects. Sens. Actuators B.

[6] Kim Y.S., Hwang I.S., Kim S.J., Lee C.Y., Lee J.H. (2008). CuO nanowire gas sensors for air quality control in automotive cabin. Sens. Actuators B.

[7] Li W.Y., Xu L.N., Chen J. (2005). Co_3_O_4_ nanomaterials in lithium-ion batteries and gas sensors. Adv. Funct. Mater..

[8] Nguyen H., Elsafty S.A. (2011). Meso-and macroporous Co_3_O_4_ nanorods for effective VOC gas sensors. J. Phys. Chem. C.

[9] Hu X., Yu J.C., Gong J., Li Q., Li G. (2007). α-Fe_2_O_3_ nanorings prepared by a microwave-assisted hydrothermal process and their sensing properties. Adv. Mater..

[10] Dirksen J.A., Duval K., Ring T.A. (2001). NiO thin-film formaldehyde gas sensor. Sens. Actuators B.

[11] Waitz T., Wagner T., Sauerwald T., Kohl C.D., Tiemann M. (2009). Ordered mesoporous In_2_O_3_: Synthesis by structure replication and application as a methane gas sensor. Adv. Funct. Mater..

[12] Li X.L., Lou T.J., Sun X.M., Li Y.D. (2004). Highly sensitive WO_3_ hollow-sphere gas sensors. Inorg. Chem..

[13] Garciasanchez R.F., Ahmido T., Casimir D., Baliga S., Misra P. (2013). Thermal effects associated with the Raman spectroscopy of WO_3_ gas-sensor materials. J. Phys. Chem. A.

[14] Wang Y., Shi J.C., Cao J.L., Sun G., Zhang Z.Y. (2011). Synthesis of Co_3_O_4_ nanoparticles via the CTAB-assisted method. Mater. Lett..

[15] Patil D., Patil P., Subramanian V., Joy P.A., Potdar H.S. (2010). Highly sensitive and fast responding CO sensor based on Co_3_O_4_ nanorods. Talanta.

[16] Barreca D., Bekermann D., Comini E., Devi A., Fischer R.A., Gasparotto A., Gavagnin M., Maccato C., Sada C., Sberveglieri G. (2011). Plasma enhanced-CVD of undoped and fluorine-doped Co_3_O_4_ nanosystems for novel gas sensors. Sens. Actuators B.

[17] Zhang L.Q., Gao Z.F., Liu C., Zhang Y.H., Tu Z.Q., Yang X.P., Yang F., Wen Z., Zhu L.P., Liu R. (2015). Synthesis of TiO_2_ decorated Co_3_O_4_ acicular nanowire arrays and their application as an ethanol sensor. J. Mater. Chem. A.

[18] Novoselov K.S., Geim A.K., Morozov S.V., Jiang D., Zhang Y., Dubonos S.V., Grigorieva I.V., Firsov A.A. (2004). Electric field effect in atomically thin carbon films. Science.

[19] Gueorguiev G.K., Broitman E., Furlan A., Stafström S., Hultman L. (2009). Dangling bond energetics in carbon nitride and phosphorus carbide thin films with fullerene-like and amorphous structure. Chem. Phys. Lett..

[20] Broitman E., Gueorguiev G.K., Furlan A., Son N.T., Gellman A.J., Stafström S., Hultman L. (2008). Water adsorption on fullerene-like carbon nitride overcoats. Thin Solid Films.

[21] Wang Y., Cao J.L., Qin C., Zhang B., Sun G., Zhang Z.Y. (2017). Synthesis and enhanced ethanol gas sensing properties of the g-C_3_N_4_ nanosheets-decorated tin oxide flower-like nanorods composite. Nanomaterials.

[22] Wang D.H., Hu Y., Zhao J.J., Zeng L.L., Tao X.M., Chen W. (2014). Holey reduced graphene oxide nanosheets for high performance room temperature gas sensing. J. Mater. Chem. A.

[23] Cao J.L., Qin C., Wang Y., Zhang H.L., Zhang B., Gong Y.X., Wang X.D., Sun G., Bala H., Zhang Z.Y. (2017). Synthesis of g-C_3_N_4_ nanosheet modified SnO_2_ composites with improved performance for ethanol gas sensing. RSC Adv..

[24] Chen N., Li X.G., Wang X.Y., Yu J., Wang J., Tang Z.N., Akbar S.A. (2013). Enhanced room temperature sensing of Co_3_O_4_-intercalated reduced graphene oxide based gas sensors. Sens. Actuators B.

[25] Cao J.L., Qin C., Wang Y., Zhang B., Gong Y.X., Zhang H.L., Sun G., Bala H., Zhang Z.Y. (2017). Calcination Method Synthesis of SnO_2_/g-C_3_N_4_ Composites for a High-Performance Ethanol Gas Sensing Application. Nanomaterials.

[26] Kessler F.K., Zheng Y., Schwarz D., Merschjann C., Schnick W., Wang X. (2017). Functional carbon nitride materials-design strategies for electrochemical devices. Nat. Rev. Mater..

[27] Miller T.S., Jorge A.B., Suter T.M., Sella A., Corà F., Mcmillan P.F. (2017). Carbon nitrides: Synthesis and characterization of a new class of functional materials. Phys. Chem. Chem. Phys..

[28] Ding L.J., Zhao M.G., Fan S.S., Ma Y., Liang J.G., Wang X.T., Song Y.W., Chen S.G. (2016). Preparing Co_3_O_4_ urchin-like hollow microspheres self-supporting architecture for improved glucose biosensing performance. Sens. Actuators B.

[29] Shaalan N.M., Rashad M., Moharram A.H., Abdelrahim M.A. (2016). Promising methane gas sensor synthesized by microwave-assisted Co_3_O_4_ nanoparticles. Mater. Sci. Semicond. Process..

[30] Ma T.Y., Dai S., Jaroniec M., Qiao S.Z. (2014). Metal-organic framework derived hybrid Co_3_O_4_-carbon porous nanowire arrays as reversible oxygen evolution electrodes. J. Am. Chem. Soc..

[31] Choi S.J., Ryu W.H., Kim S.J., Cho H.J., Kim I.D. (2014). Bi-functional co-sensitization of graphene oxide sheets and Ir nanoparticles on p-type Co_3_O_4_ nanofibers for selective acetone detection. J. Mater. Chem. B.

[32] Li J., Tang S.B., Lu L., Zeng H.C. (2007). Preparation of nanocomposites of metals, metal oxides, and carbon nanotubes via self-assembly. J. Am. Chem. Soc..

[33] Chatterjee S.G., Chatterjee S., Ray A.K., Chakraborty A.K. (2015). Graphene–metal oxide nanohybrids for toxic gas sensor: A review. Sens. Actuators B.

[34] Liang Y.Y., Li Y.G., Wang H.L., Zhou J.G., Wang J., Regier T., Dai H.J. (2011). Co_3_O_4_ nanocrystals on graphene as a synergistic catalyst for oxygen reduction reaction. Nat. Mater..

[35] Kim H.J., Lee J.H. (2014). Highly sensitive and selective gas sensors using p-type oxide semiconductors: Overview. Sens. Actuators B.

[36] Wang C., Zhu J.W., Liang S.M., Bi H.P., Han Q.F., Liu X.H., Wang X. (2014). Reduced graphene oxide decorated with CuO–ZnO hetero-junctions: Towards high selective gas-sensing property to acetone. J. Mater. Chem. A.

[37] Mehrabadi Z.S., Ahmadpour A., Shahtahmasebi N., Mohagheghi M.M.B. (2011). Synthesis and characterization of Cu doped cobalt oxide nanocrystals as methane gas sensors. Phys. Scr..

[38] Cao J.L., Gong Y.X., Wang Y., Zhang B., Zhang H.L., Sun G., Bala H., Zhang Z.Y. (2017). Cocoon-like ZnO decorated graphitic carbon nitride nanocomposite: Hydrothermal synthesis and ethanol gas sensing application. Mater. Lett..

[39] Wang Y., Huang J., Cao J.L., Li G.J., Zhang Z.Y. (2017). Cobalt oxide decorated flower-like g-C_3_N_4_ hybrid nanomaterials for carbon monoxide oxidation. Surf. Rev. Lett..

